# Severely Disseminated Kaposi Sarcoma after ABO-Incompatible Kidney Transplantation Treated Successfully with Paclitaxel and Gemcitabine Combined with Hemodialysis

**DOI:** 10.1155/2019/8105649

**Published:** 2019-12-11

**Authors:** Tobias Bomholt, Anders Krarup-Hansen, Martin Egfjord, Søren Schwartz Sørensen, Niels Junker

**Affiliations:** ^1^Department of Nephrology, Rigshospitalet, Copenhagen, Denmark; ^2^Department of Oncology, Herlev Hospital, Herlev, Denmark

## Abstract

Kaposi Sarcoma (KS) is driven by human herpes virus 8 causing vascular proliferation which is induced by loss of immune function most often due to HIV or immunosuppressants. KS occurs with increased incidence in kidney transplant recipients, but rarely is disseminated. We report a 64-year-old male who developed severely disseminated KS 5 months after ABO-incompatible kidney-transplantation. No guidelines for chemotherapy exist in this case and reduced kidney function and impaired immune system complicates the use of systemic chemotherapy in kidney transplant recipients. A combination of paclitaxel and gemcitabine followed by two days of hemodialysis treatment was chosen since paclitaxel can be given in full dose independently of kidney function and gemcitabine is metabolised to 2′,2′-difluorodeoxyuridine which is found to be highly dialysable. The present treatment was well tolerated by the patient with one episode of leukopenia and elevated alanine transaminase during treatment which resolved. There were no serious adverse events and the patient obtained a complete remission verified by Positron Emission Tomography CT after ending chemotherapy and at one-year follow up.

## 1. Introduction

Kaposi Sarcoma (KS) is a viral induced cancer where infection with human herpes virus 8 (HHV-8) is considered the etiological agent [1]. Loss of immune function most often due to HIV or immunosuppressants impairing T-cell reactivity, increases the risk of viral reactivation and KS occurrence [2, 3]. Due to an increase in the prevalence of organ transplanted recipients, mainly kidney transplanted recipients, and the use of immunosuppressive therapy the risk of iatrogenic KS has increased. Among kidney-transplant recipients, a large multicentre study found the incidence of KS to be 0.69% during a median follow-up period of 24 months [4]. KS was mainly cutaneous and rarely showed visceral involvement with an incidence of 0.6% and 0.09%, respectively [4]. A high incidence of KS in kidney-transplant recipients is reported in numerous studies compared with the general population suggesting a role for immunosuppression in the development of the disease [5, 6].

For kidney transplant recipients with only cutaneous involvement of KS, complete remission has been obtained by conversion from cyclosporine to sirolimus [7]. For KS with visceral involvement, no case series or treatment guidelines exist for kidney transplant recipients. In HIV-related KS, systemic chemotherapy is generally indicated in rapidly progressive or disseminated disease. Liposomal anthracyclines and taxanes are proven active drugs in this setting [8].

Systemic chemotherapy in kidneytransplant recipients is complicated due to a reduction in renal clearance and altered metabolism of drugs. There is also a high risk of adverse events since the immune system is already compromised by immunosuppressive drugs. Therefore, the chemotherapies of choice should consider the potential risks of unknown renal clearance and potential risk of side effects as seen for liposomal anthracyclines [8, 9]. Taxanes alone or in combination therapy could represent a more manageable alternative in relation to decreased renal function [8].

## 2. Case Report

**Figure 1:**
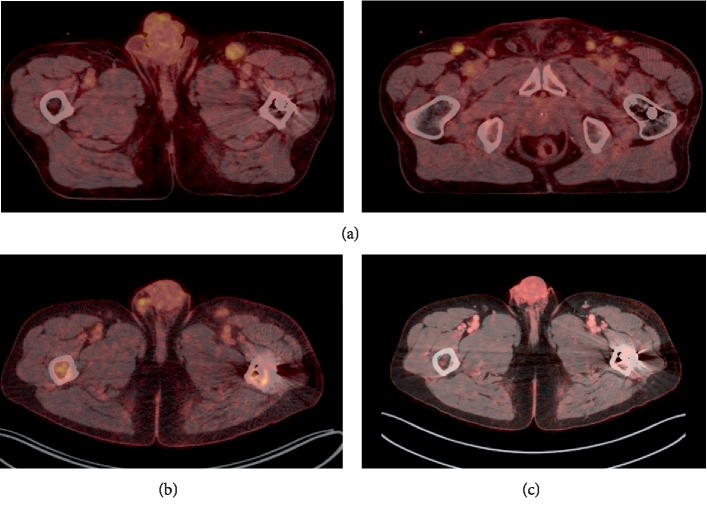
(a) PET-CT at baseline showing an enlarged lymph node in the left and right groin measuring 2.7 × 1.9 cm and 1.4 × 1.6 cm, respectively, with increased metabolic activity. Enlarged and hypermetabolic lymph nodes were also found in the mediastinum and the left side of the retroperitoneum. (b) PET-CT after course 10 showing lymph nodes in the left groin with normal size and metabolic activity. Rest of the PET-CT showed complete remission of other tumour sites. (c) PET-CT after 15 months at follow up with no signs of relapse.

A 64-year-old male developed severely disseminated KS five months after ABO-incompatible kidney-transplantation. The patient had end-stage renal disease due to autosomal dominant polycystic kidney disease and was on peritoneal dialysis for eight months prior to transplantation. Per protocol, the patient was treated with a single dose rituximab (375 mg/m^2^) and intravenous (i.v.) immunoglobulin therapy (IVIG) before transplantation and induction therapy was i.v. basiliximab and methylprednisolone (MP). Posttransplantation immunosuppression consisted of prednisolone, tacrolimus (Tac), and mycophenolatemofetil (MMF). One week after transplantation, the patient was treated for borderline rejection with three days of i.v. MP (250 mg/day). At discharge, serum creatinine was 1.6 mg/dl and immunosuppression was tapered in the outpatient clinic. Five months after transplantation, the patient developed purple-red elements on the skin of the forehead, nose, and penis. Two months after presentation of skin elements, the patient was admitted and enlarged lymph nodes in both groins were found. Biopsies from both skin and lymph nodes revealed KS with tumour growth through the capsule of the lymph node. A Positron Emission Tomography CT (PET-CT) revealed pathologically enlarged and metabolically active lymph nodes in the mediastinum, left side of the retroperitoneum, and both groins as seen in Figure 1(a). The patient tested positive for HHV-8 and negative for HIV. Ten cycles of paclitaxel (30 mg) and gemcitabine (304 mg) were given on days 1 and 8, with hemodialysis performed the following two days after chemotherapy to remove metabolites of gemcitabine. Paclitaxel and gemcitabine doses were increased over the treatment period to a final dose of 138 mg (approximately 80 mg/m^2^) and 1416 mg (approximately 800 mg/m^2^), respectively. Immunosuppression was initially changed by discontinuing MMF and switching from Tac to everolimus (Eve). Due to the severity of the disease Eve was also stopped and prednisolone 5 mg/day was given as monotherapy. Throughout the treatment period, the patient had great variation in creatinine due to infections and urinary tract obstruction with creatinine ranging between 1.7 and 6.2 mg/dl. PET-CT performed after courses 4 and 6 and 8 showed regression of all lymph node enlargements and complete remission of KS after course 10. Hemodialysis was discontinued after termination of chemotherapy and the patient then maintained a creatinine of 1.6 mg/dl. A PET-CT 15 months later showed continuous complete remission.

## 3. Discussion

Severely disseminated KS in kidney-transplant recipients is a rare condition and no clear recommendations on chemotherapy treatment exist. This patient had received an ABO-incompatible kidney-transplant with a protocol including rituximab and IVIG, and received furthermore i.v. MP for borderline rejection. In line with the historical data indicating an association with decreased immune function and KS severity, this increased immunosuppressive treatment may explain the rapid progression of KS in this patient. First-line treatment with liposomal anthracyclines was not chosen due to the uncertain pharmacokinetics imposing a high risk of side effects or insufficient treatment if the dose is reduced [9]. A combination of paclitaxel and gemcitabine followed by two days of hemodialysis treatment was preferred instead due to a more favourable pharmacokinetic profile: paclitaxel can be given in full dose independently of kidney function and gemcitabine is metabolised to 2′,2′-difluorodeoxyuridine (dFdU) which is found to be highly dialysable. This makes it a safe treatment option when performing hemodialysis after chemotherapy treatment to remove the metabolites [10]. The present treatment was well tolerated by the patient: the patient had one episode of leukopenia (1.3 × 10^9^) and elevated alanine transaminase (maximum of 133 U/L) during treatment which resolved. There were no serious adverse events and the patient obtained a complete remission on PET-CT as seen in Figure 1(b).

Reduction in immunosuppression remains a key part of treatment for KS. One option was to remove the renal graft to allow for a complete tapering of immunosuppressive drugs. However, since PET-CT revealed early KS regression, the renal graft was not removed, but immunosuppression was reduced to a minimum of 5 mg of prednisolone; a dose still maintained to minimize risk of KS recurrence and without any signs of transplant rejection. At 15 months posttreatment follow up, the patient is KS recurrence free as shown by PET-CT in Figure 1(c), doing well (Performance status according to Eastern Cooperative Oncology Group = 0) without any treatment related adverse events, and has maintained renal graft function with creatinine 15 months after ending treatment at 1.8 mg/dl.
